# Bacterial Cellulose: A Versatile Chiral Host for Circularly Polarized Luminescence

**DOI:** 10.3390/molecules24061008

**Published:** 2019-03-13

**Authors:** Chen Zou, Dan Qu, Haijing Jiang, Di Lu, Xiaoting Ma, Ziyi Zhao, Yan Xu

**Affiliations:** State key Laboratory of Inorganic Synthesis and Preparative Chemistry, Jilin University 2699 Qianjin Street, Changchun 130012, China; chris_zou@163.com (C.Z.); qudan0311@163.com (D.Q.); hjjiang2019@163.com (H.J.); mimituer@163.com (D.L.); MxtJLU@126.com (X.M.); z18844112609@163.com (Z.Z.)

**Keywords:** bacterial cellulose, circularly polarized luminescence, chiral host, ambient conditions, sustainable materials

## Abstract

Materials capable of circularly polarized luminescence (CPL) have attracted considerable attention for their promising potential applications. Bacterial cellulose (BC) was characterized as having a stable right-handed twist, which makes it a potential chiral host to endow luminophores with CPL. Then, the CPL-active BC composite film was constructed by simply impregnating bacterial cellulose pellicles with dilute aqueous solutions of luminophores (rhodamine B, carbon dots, polymer dots) and drying under ambient conditions. Simple encapsulation of luminophores renders BC with circularly polarized luminescence with a dissymmetry factor of up to 0.03. The multiple chiral centers of bacterial cellulose provide a primary asymmetric environment that can be further modulated by supramolecular chemistry, which is responsible for its circular polarization ability. We further demonstrate that commercial grade paper may endow luminophores with CPL activity, which reifies the universality of the method.

## 1. Introduction

Materials capable of circularly polarized luminescence (CPL) have attracted considerable attention for their promising potential applications in display technologies, biosensing, chiral catalysis, diffraction-free patterning, and telecommunication [1,2,3,4,5,6,7,8,9,10,11]. One kind of conventional strategy of generating CPL is to use complex achiral luminophores with chiral supramolecular assemblies [12,13,14,15,16,17]. The homochirality, specifically left or right handedness, of biological structures is one of the most fascinating phenomena in nature [18]. That means there are lots of promising natural chiral host matrices for generating CPL. For example, natural amino acids and natural sugars exist predominantly as left-handed (LH) and right-handed (RH) enantiomers, respectively. Nam et al. used amino acids and peptides to synthesize chiral gold nanoparticles and control the handedness and chiral plasmonic resonance of the nanoparticles [19]. Chiral nanostructures of DNA–Au ensembles displayed high polarization rotation leading to strong chiroptical activity [5]. Emergence of CPL in a multiple-component dendron hydrogel system containing achiral quantum dots was attributed to chirality transfer [20]. Most contemporary CPL-active materials display luminescence dissymmetry factors in the range of 10^−3^–10^−2^, and they are usually synthesized by tedious procedures using costly materials and often causing a negative impact on the environment which could be alleviated, to some extent, by using natural products. 

Cellulose is the most earth-abundant renewable polymer that is widely distributed in higher plants as a basic structural component and in a few marine animals such as tunicates [21]. Cellulose is also produced by some microorganisms, such as *Acetobacter xylinum*, *A. Hansenii*, and *A. pasteurianus*, as first discovered by A. J. Brown in 1886 and known as bacterial cellulose (BC) [22]. Cellulose, regardless of its source, is a high-molecular-weight homopolymer of β-1,4-linked anhydro-D-glucose units in which every unit is twisted 180° with respect to its neighbors, and the repeat segment is characterized as a glucose dimer. Bacterial cellulose is extracellularly synthesized as a protective envelope around cells. When cultured under static conditions, it forms into a pellicle-shaped hydrogel. When dried under ambient conditions, it gives a transparent sheet of bacterial cellulose paper if the shrinkage across the sheet is restricted. The water retention value of bacterial cellulose is 1000% in contrast to 60% of plant cellulose. Bacterial cellulose displays unique properties including high chemical purity, great tensile strength, hydrophilicity, high crystallinity, an ultrafine fiber network architecture, and biocompatibility. The Young’s modulus across the plane of bacterial cellulose is as high as 15 GPa, owing to the unique supramolecular structures in which fibrils are bound tightly by inter- and intrachain hydrogen bonding [23]. Due to its many unique properties, bacterial cellulose has been widely used in the food industry, in the biomedical field, and as a functional additive for higher-value commercial products. Bacterial cellulose-based advanced materials display potential applications in biomedicine, environmental protection, and new energy materials [21,24]. It was not until recently that the microfibrils of bacterial cellulose were characterized as a stable right-handed twist [25,26], which makes bacterial cellulose a potential chiral host to endow luminophores with CPL. So far, the circular polarization potential of bacterial cellulose, to the best of our knowledge, has not been exploited. 

Herein, we report that transparent bacterial cellulose paper encapsulating luminophores enabled left-handed CPL with a dissymmetry factor of up to 0.03. The CPL-active paper was constructed by simply impregnating bacterial cellulose pellicles with dilute aqueous solutions of luminophores (rhodamine B, carbon dots, polymer dots) and drying under ambient conditions. The bacterial cellulose-rhodamine B paper (BC-RhB) displayed a predominant left-handedness with more complex chiral information than the pristine bacterial cellulose (BC), likely attributed to the supramolecular assembly of BC and RhB, leading to chirality transfer. The right-handed twisted nanofibers of bacterial cellulose were not the key contributor to the CPL activity of BC-RhB. The BC-RhB paper displayed birefringence patterns, which may point to the presence of long-range structural anisotropy. We further demonstrate that commercial grade paper may endow luminophores with CPL activity.

## 2. Results and Discussion

### 2.1. Bacterial Cellulose-Luminophore Composited Films Exhibited CPL Property

**Scheme 1:**
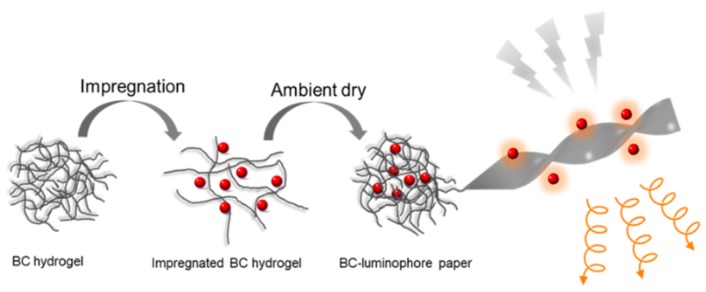
Bacterial cellulose (BC)-luminophore paper prepared by impregnation and ambient drying, and its potential for circularly polarized luminescence.

**Figure 1:**
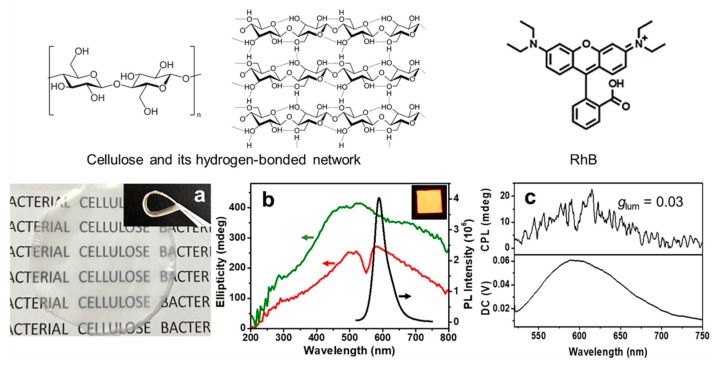
(**a**) Photograph of BC-RhB (rhodamine B) paper showing its good transparency and flexibility (inset). (**b**) Circular dichroism (CD) spectra of BC-RhB (red line) and pristine BC-paper (green line) (left vertical axis) and photoemission spectrum of BC-RhB (right vertical axis). Inset: Photograph of BC-RhB under 365 nm irradiation. (**c**) Circularly polarized luminescence (CPL) spectrum of BC-RhB (RhB loading = 0.67 μg/mg, paper thickness = 55 μm).

The bacterial cellulose-luminophore paper (BC-paper) was prepared by impregnation in luminophore solutions followed by drying under ambient conditions (Scheme 1, Figure 1a). Its thickness, which determines the transparency, can be easily tuned by controlling the culture time of bacterial cellulose biosynthesis [27]. The CPL ability of BC was examined using rhodamine B-encapsulated bacterial cellulose paper (thickness = 55 μm, transparency = 50%), denoted as BC-RhB. As shown in Figure 1b, BC-RhB appeared homogeneous and orange under 365 nm irradiation, suggesting well-dispersed RhB. The photoemission quantum yield and lifetime were 33.5% and 22.83 ns, respectively. BC-RhB displayed broad positive circular dichroism (CD) signals in the range from 200 to 800 nm (Figure 1b red line). Notably, the CD signals of BC-RhB were weaker in intensity and displayed two peak maxima at 510 and 580 nm instead of one peak maxima at 515 nm for the pristine BC (Figure 1b green line), which may due to the induced CD phenomenon. This suggests that the supramolecular assembly of BC and RhB causes chirality transfer, leading to a change in the asymmetric environment in BC-RhB. In addition, the linear dichroism (LD) spectra of BC were also recorded to exclude possible LD artifacts. As manifested in Figure S1, the contribution of the LD effect was weak enough to be negligible.

To evaluate the CPL ability of BC-RhB, CPL spectra were recorded on a JASCO CPL-200 spectrometer with an in-line excitation/emission configuration to allow for determination of the photoluminescence dissymmetry factors under artifact-free conditions [28], and the film thickness was controlled to approximately 55 μm. Consecutive measurements yielded reproducible results and revealed the optical stability of BC-RhB under irradiation. The CPL degree can be expressed using the dissymmetry factor *g*_lum_ = 2(*I*_L_ − *I*_R_)/(*I*_L_ + *I*_R_), where *I*_L_ and *I*_R_ are the intensities of left- and right-handed CPL, respectively [29]. It was calculated by *g*_lum_ = 2(ellipticity/(32980/ln10))/total fluorescence intensity at the CPL extremum. Indeed, BC-RhB (thickness = 55 μm, RhB loading = 0.67 μg/mg) enabled L-CPL with the maximum *g*_lum_ value of 0.03 (Figure 1c). The magnitude of circular polarization at the ground state, the absorptive dissymmetry factor, is defined as g_abs_ = 2(ε_L_ − ε_R_)/( ε_L_ + ε_R_), where ε_L_ and ε_R_ refer to the extinction coefficients for left- and right-handed circularly polarized light, respectively. Experimentally, the value of g_abs_ is defined as g_abs_ = (ellipticity/32980)/absorbance at the CD extremum [30]. The g_abs_ of BC-RhB was 0.018, which was in the same order of magnitude as the *g*_lum_ value.

### 2.2. CPL Property Effected by Film Thickness and Loading Amount

**Figure 2:**
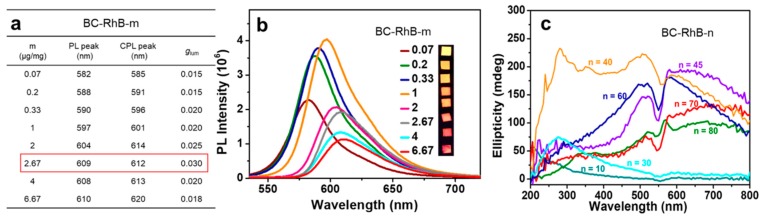
(**a**) BC-RhB-m enabling L-CPL of varied peak wavelengths and *g*_lum_ values (m refers to RhB solution concentration in μg/mg, paper thickness = 40 μm). (**b**) Corresponding photoluminescence spectra and photographs of BC-RhB-m. (**c**) CD spectra of BC-paper-n (n refers to paper thickness in μm, RhB loading = 0.67 μg/mg).

To further establish understanding of the CPL ability, two series of BC-RhB were prepared. BC-RhB-m was characterized by varied RhB loading at the paper thickness of 40 μm, and BC-RhB-n was characterized by varied paper thickness at the RhB loading of 0.67 μg/mg. As shown in Figure 2a, BC-RhB-m enabled exclusively L-CPL with varied *g*_lum_ values and peak wavelengths with the RhB loading change. The L-CPL with the maximum *g*_lum_ value of 0.03 at 612 nm was found in BC-RhB-2.67. Photoemission spectral measurements showed interesting emission features with the RhB loading change in BC-RhB-m (Figure 2b). BC-RhB-1 displayed the highest photoemission intensity with the peak maxima at 597 nm and BC-RhB-2.67 displayed much lower photoemission intensity than BC-RhB-1 with the peak maxima at 609 nm, likely attributed to aggregation-induced fluorescence quenching. Notably, the induced CD was observed clearly when increasing the RhB loading amount (Figure S2a), which confirmed that the supramolecular assembly of BC and RhB causes chirality transfer leading to the change in the asymmetric environment in BC-RhB which we discussed above. The two main peaks in the CD spectra of BC-RhB-m, at 560 and 525 nm, correspond to the main absorbance peak of RhB at 554 nm and the minor peak at 514 nm (Figure S2b), which were red shifted along with the increase of the loading of RhB. Similar to BC-RhB-m, BC-RhB-n enabled exclusively L-CPL with varied peak wavelength and *g*_lum_ values, among which L-CPL with the highest *g*_lum_ value of 0.03 at 596 nm was found in BC-RhB-55 (Figure S3). An extraordinary phenomenon is displayed in that BC-RhB-m yielded drastically different CD spectra with the change in paper thickness (Figure 2c). BC is a homopolysaccharide with abundant hydroxyl groups containing a number of chiral centers, and RhB is a water-soluble organic chloride salt (Figure 1). The complexity in the CD signals of BC-RhB may be, to some extent, attributed to the supramolecular interactions involving BC and RhB, leading to a change in the asymmetric environment of the chiral centers. These observations point to the local mechanism that is responsible for the L-CPL of BC-RhB. The BC-luminophore papers enabled L-CPL with a dissymmetry factor in the range of 10^−3^–10^−2^ (Figure 2a, Figure S3).

### 2.3. Structural Anisotropy in BC

**Figure 3:**
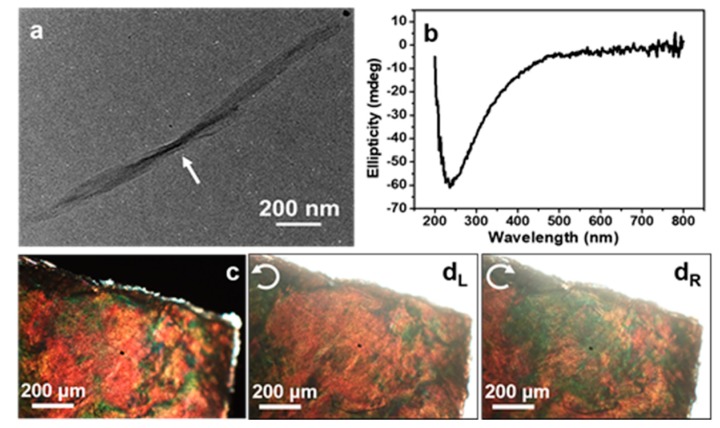
(**a**) TEM image of CNC derived from bacterial cellulose. (**b**) CD spectrum of 3 wt % aqueous solution of the bacterial cellulose-derived CNC. (**c**) Polarizing optical microscope (POM) image of BC (thickness = 55 μm) under crossed polarizers. Transmitted optical images of BC-paper (thickness = 55 μm) by 400–700 nm circularly polarized light: (**d_L_**) left and (**d_R_**) right.

Towards a further understanding of the CPL ability of BC-RhB, cellulose nanocrystals derived from BC by acid hydrolysis were characterized. The right-handed twisted morphology of the cellulose nanocrystals was observed in the transmission electron microscopy (TEM) image (Figure 3a). A negative CD signal with a peak maxima at 235 nm was shown on the CD spectrum in keeping with the right-handed twisted morphology of the cellulose nanocrystals (Figure 3a,b). In view of the fact that BC-RhB enabled exclusively L-CPL, this points to the insignificant contribution of cellulose nanocrystals to the CPL properties of BC-RhB (Figure 1c, Figure 2a, Figure S3c). The BC-paper was further characterized using polarized optical microscopy (POM). As shown in Figure 3c, BC displayed strong birefringence patterns of red and green under crossed polarizers, revealing long-range structural anisotropy. Transmitted optical images of the same BC film were photographed using 400–700 nm left circularly polarized light (LCP) and right circularly polarized light (RCP) as a backlight. The predominant transmitted color of red under LCP (Figure 3d_L_) became green under RCP (Figure 3d_R_). This observation was in agreement with the POM imaging, pointing to a long-range structural anisotropy in BC.

### 2.4. The Universality of the Method

**Figure 4:**
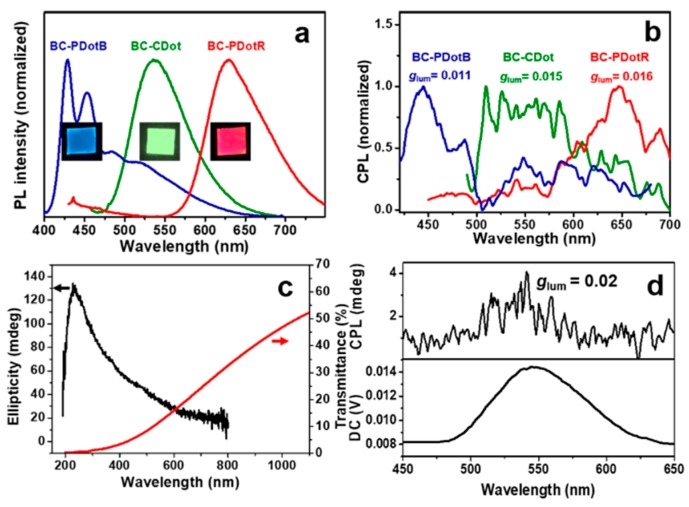
(**a**) Photoluminescence spectra of BC-luminophore papers and corresponding photographs under 365 nm irradiation. (**b**) CPL spectra of the BC-luminophore papers. (**c**) CD (left vertical axis) and UV–vis transmission spectra (right vertical axis) of cellulose-CDot paper. (**d**) CPL spectrum of the cellulose-CDot paper.

To verify the applicability of BC as a general chiral host to bestow luminophores with circularly polarized luminescence, the CPL ability of BC-luminophore was further examined using carbon dots (CDot), poly(9,9-dihexylfluorenyl-2,7-diyl) (PDotB), and poly(9,9-dioctylfluorene)-co-(4,7-di-2-thienyl-2,1,3-benzothiadiazole) (PDotR). PDotB and PDotR were polymer dots with blue- and red-emitting colors, respectively, and they were negatively charged nanoparticles with an average particle size of 20 and 30 nm, and zeta potential values of −35.1 and −40.2 mV, respectively (Figure S4). CDot was positively charged with an average particle size of 12.5 nm (Figure S5). The photoemission spectra and fluorescent images of BC-luminophore showed bright emitting colors across the visible spectrum from blue to red (Figure 4a). The photoemission peak maxima of BC-luminophore were very similar to the corresponding dilute solutions (Figure S6). Surprisingly, simple encapsulation of luminophores in BC led to L-CPL with the maximum *g*_lum_ = 0.011 at 445 nm for BC-PDotB, *g*_lum_ = 0.015 at 552 nm for BC-CDot, and *g*_lum_ = 0.016 at 645 nm for BC-PDotR (Figure 4b). The L-CPL peak maxima enabled by the BC-luminophore paper coincided with the peak maxima of the photoemission band of PDotB, CDot, and PDotR, suggesting that L-CPL originates from a local mechanism due to the asymmetric environment. It is worth noting that BC-luminophore papers displayed L-CPL across the entire visible spectrum with luminescence dissymmetry factors in the range of 10^−3^–10^−2^, comparable to or even greater than many synthetic CPL-active organic materials. Our experimental results reveal that BC avails a powerful and versatile platform to endow wide-ranging luminophores of varied sizes and electrostatic states with circularly polarized luminescence.

Regardless of its source, cellulose is a homopolymer of β-1,4-linked anhydro-D-glucose units that interacts strongly forming a hydrogen-bonded network (Figure 1). To investigate the CPL potential of cellulose, we chose a commercial grade paper and impregnated it in a dilute aqueous solution of CDot based on Scheme 1, giving rise to cellulose-CDot paper. It displayed good transparency and a positive CD signal (Figure 4c). The CPL spectrum recorded on the cellulose-CDot paper (thickness = 30 μm, CDot loading = 0.5 μg/mg) was shown in Figure 4d. Indeed, cellulose-CDot enabled L-CPL with the maximum *g*_lum_ value of 0.02 at 540 nm, coinciding with the photoemission peak maxima of CDot (Figure S6).

BC provides a versatile chiral host for circularly polarized luminescence. Compared with artificial composite materials, natural products, such as bacterial cellulose, neither require the tedious synthesis nor infringe the principles of cost reduction and green chemistry. However, the mechanism of BC generating CPL is not completely determined yet and needs further research. Additionally, the asymmetry factor of BC-generated CPL is still not comparable to some other materials, for example, chiral photonic cellulose films whose asymmetry factor can be up to 0.87 [12]. BC is produced by living bodies, which makes it hard to control as precisely as a chemical synthesis reaction. However, its intrinsic properties, such as great mechanical properties, biocompatibility, and ease of post-synthetic modification, still show BC has great potential applications as a CPL-generating material. 

## 3. Materials and Methods

### 3.1. Materials

*Acetobacter xylinum* strains were donated by Tianjin University. Glucose, peptone, and yeast extract were purchased from Oxoid Company. Disodium phosphate, sodium hydroxide, and citric acid were purchased from Beijing Chemical Works. Sulfuric acid was purchased from Beijing Chemical Industry Group Co. Ltd. Rhodamine B (RhB) was purchased from Acros Reagent Company. CDot was donated by Jilin University, which was synthesized as previously reported [31]. Poly(9,9-dihexylfluorenyl-2,7-diyl) (PDHF, 0.0153 wt %, PDotB) and poly(9,9-dioctylfluorene)-co-(4,7-di-2-thienyl-2,1,3-benzothiadiazole) (PF-5DTBT, 0.0121 wt %, PDotR) were prepared according to reported procedures [32]. Commercial paper was purchased from Anhui Shanyang Paper Industry Co. Ltd. All reagents were used as received without further purification.

### 3.2. Preparation of BC 

*Acetobacter xylinum* inoculums were added into sterilized culture media (2.5% (*w/v*), glucose, 1% (*w/v*) peptone, 0.75% (*w/v*) yeast extract, 1% (*w/v*) disodium phosphate, adjusted pH to 5 by citric acid) and statically incubated at 30 °C for days until the desired BC thickness was achieved. The BC pellicles were treated for 2 h in 4 wt % NaOH solution at 80 °C, then rinsed with distilled water until rinses displayed neutral measured pH values. The purified BC pellets were dried under ambient conditions and cut into 1 cm^2^ small pieces.

### 3.3. Preparation of BCNC 

The BC pulp was directly hydrolyzed using H_2_SO_4_ as described [33]. In short, the purified BC pellicles were cut into small cubes and homogenized at 5000 rpm for 2 min using a homogenizer without any additional water added, which resulted in a cellulosic pulp. Cellulosic pulp (5.0 g) was hydrolyzed in aqueous H_2_SO_4_ solutions (20 mL) of 60% (*v/v*) at 35 °C for 3 h with stirring at 400 rpm. The cellulose suspension was then diluted with cold distilled water to halt the hydrolysis reaction. The resultant white suspension was centrifuged at 11,000 rpm for 10 min to collect the hydrolyzed products, followed by dialysis with regenerated cellulose dialysis tubing (8000–14,000 MWCO, Thermo Scientific, Waltham, MA, USA) against distilled water until the pH reached a neutral value. Then, sonication was performed on the BC nanocrystal suspension using a Sonics Vibra-Cell (VCX-750, Sonics & Materials. Inc, Newtown, CT, USA) equipped with a 13 mm probe for 5 min, within an ice bath. 

### 3.4. Preparation of BC-X Composite Films 

The purified BC hydrogel pellets were dried under ambient conditions to form films, and then cut into 1 cm^2^ square pieces. We impregnated each small piece of bacterial cellulose film in a stoichiometric amount of dilute aqueous solutions of luminophores. Due to its excellent swelling properties, the tight BC film would recover to the hydrogel form immediately upon coming into contact with solution. Luminophores could easily get into pores between cellulose fibers along with solvent. Then, the composite pieces were dried on polystyrene Petri dishes under ambient conditions again after the luminophores had been trapped within. By varying the luminophores, composite films with different fluorescent molecules were prepared, denoted as BC-X, where X refers to the kind of molecule (X = PDotB, CDot, PDotR, and RhB). The RhB loading amount was 0.67 μg/mg and the others were all 1 μg/mg. The same processing was performed with commercial paper.

### 3.5. Preparation of BC-RhB-m Composite Films

We impregnated each 1 cm^2^ 40 μm-thick BC piece in a stoichiometric amount of RhB solution, then the BC pieces were dried on polystyrene Petri dishes under ambient conditions. By varying the RhB loading, composite films with different RhB loadings were prepared, denoted as BC-RhB-m, where m was the weight permillage of RhB in BC-RhB-m (m = 0.07, 0.2, 0.33, 1, 2, 2.67, 4, and 6.67 μg/mg). 

### 3.6. Preparation of BC-RhB-n Composite Films

We impregnated each 1 cm^2^ BC piece in a stoichiometric amount of RhB solution, then the BC pieces were dried on polystyrene Petri dishes under ambient conditions. By varying the thickness of the BC, composite films with different BC thicknesses were prepared, denoted as BC-RhB-n, where n was the thickness of BC-RhB-n (n = 10, 30, 40, 45, 55, 60, 65, 70, and 80 μm), and the loading amounts were controlled at 0.67 μg/mg.

### 3.7. Characterization

Fluorescence spectra were recorded on a FluoroMax-4 fluorescence spectrophotometer (Horiba Scientific). Transmission electron microscopy (TEM) was conducted on a FEI Tecnai G2S-Twin F20 with a field emission gun operating at 200 kV. The quantum yields and lifetime were recorded on a FLS920 fluorescence spectrometer (Edinburgh Instrument). Zeta potential was measured on Malvern Zetasizer Nano ZS90. Polarized optical microscopy (POM) was performed on Leica DM4000M versatile upright microscope. Ultraviolet–visible (UV–vis) transmission spectroscopy was conducted on a Shimadzu UV-1800 UV–vis spectrophotometer. Transmission spectra were collected by mounting free-standing films normal to the beam path. Circular dichroism (CD) and linear dichroism (LD) spectra were recorded on a BioLogic MOS-450 spectropolarimeter, and the films were mounted normal to the beam path. Circularly polarized luminescence (CPL) spectra were recorded on JASCO CPL-200 in 180° geometry. 

To exclude possible LD artifacts, CD and LD spectra were collected at every 45° (from 0° to 360°) by rotating the samples around the optical axis and, finally, average CD and LD spectra were taken for analysis. The measured LD spectra are unified as the same unit (mdeg) with CD spectra according to the following Equation:

1 mdeg = 3.298 × 10^−4^ Delta A. (1)

The LD contribution was eliminated from measured CD signal based on a reported semiempirical Equation [34]:

CD = CD_measured_ − LD × 0.02. (2)

## 4. Conclusions

In summary, we have experimentally demonstrated that the intrinsic chirality of bacterial cellulose may transform spontaneous photoemission to circularly polarized luminescence with dissymmetry factors of up to 0.03. This is realized by simple impregnation of bacterial cellulose pellicles in luminophore solutions followed by ambient drying. Our findings suggest that cellulose, regardless of its source, provides a versatile chiral host for circularly polarized luminescence. The chiral centers in a percolating network of hydrogen bonds in cellulose may be responsible for the circularly polarized luminescence, which can be further modulated, leading to a change in the properties of circularly polarized luminescence. Further work will aim at the mechanistic insight of the circularly polarized luminescence ability of cellulose and the modulation of circularly polarized luminescence through chirality transfer- and amplification-leveraging supramolecular chemistry. Such circular polarization ability may add to existing cellulose-based functional materials with additional chiroptical activity.

## Supplementary Materials

The Supplementary Materials are available online. Figure S1: CD and LD spectra of BC. Figure S2: CD spectra of BC-RhB-m and UV–vis transmission spectra of RhB solution, BC, and BC-RhB. Figure S3: Characterization of BC-RhB-n. Figure S4: TEM images of PDotB and PDotR. Figure S5: TEM images of CDot. Figure S6: PL spectra of PDotB, CDot, RhB, and PDotR solution.
