# Supplementary material for: Bacterial Cellulose: A Versatile Chiral Host for Circularly Polarized Luminescence
Source: Molecules. 2019 Mar 13;24(6):1008. doi: 10.3390/molecules24061008 (PMC6471878; doi:10.3390/molecules24061008)
Supplement: Supplementary file 1 [file molecules-24-01008-s001.pdf]

Supplementary Materials:

Communication

Bacterial Cellulose: A Versatile Chiral Host for Circularly Polarized Luminescence

Chen Zou, Dan Qu, Haijing Jiang, Di Lu, Xiaoting Ma, Ziyi Zhao and Yan Xu *

State key Laboratory of Inorganic Synthesis and Preparative Chemistry, Jilin University 2699 Qianjin Street, Changchun 130012, China; chris_zou@163.com (C.Z.); qudan0311@163.com (D.Q.); hjjiang2019@163.com (H.J.); mimituer@163.com (D.L.); MxtJLU@126.com (X.M.), z18844112609@163.com (Z.Z.)

* Correspondence: yanxu@jlu.edu.cn;

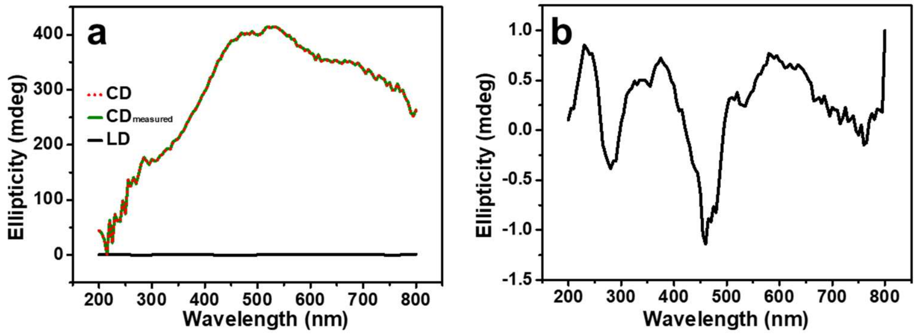

Figure S1. a) CD and LD spectra of BC. b) enlarged LD spectrum of BC.

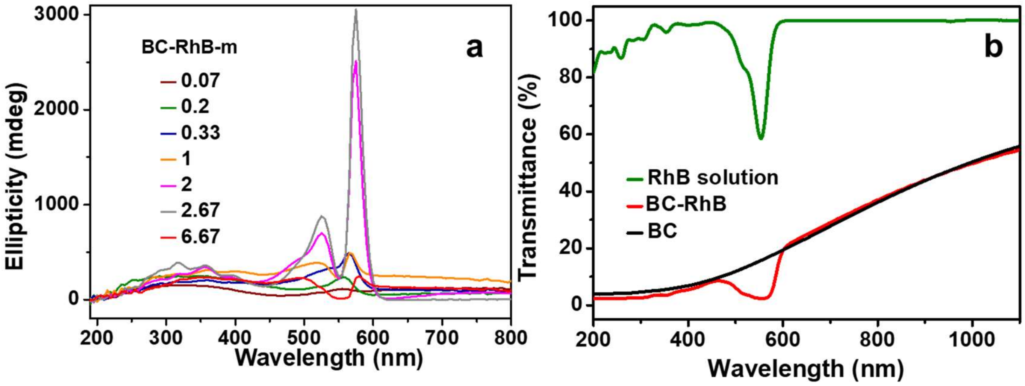

Figure S2. a) CD spectra of BC-RhB-m. b) UV-Vis transmission spectra of RhB solution, BC and BC-RhB.

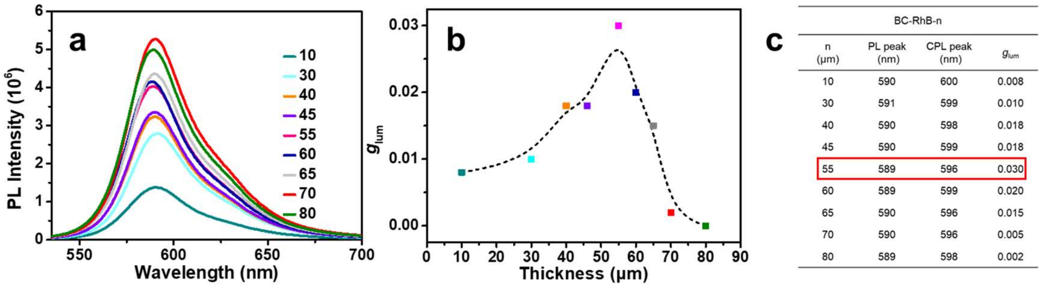

BC-RhB-n			
n (μm)	PL peak (nm)	CPL peak (nm)	g_{lum}
10	590	600	0.008
30	591	599	0.010
40	590	598	0.018
45	590	599	0.018
55	589	596	0.030
60	589	599	0.020
65	590	596	0.015
70	590	596	0.005
80	589	598	0.002

Figure S3. Characterization of BC-RhB-n. a) PL spectra. b, c) g_{lum} of BC-RhB-n.

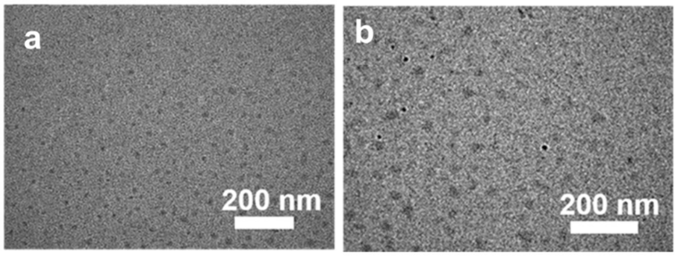

Figure S4. TEM images of a) PDotB and b) PDotR.

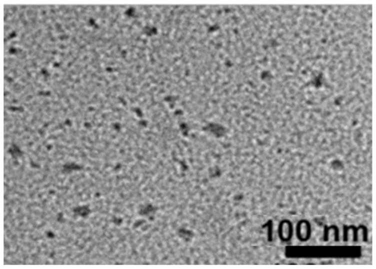

Figure S5. TEM images of CDot.

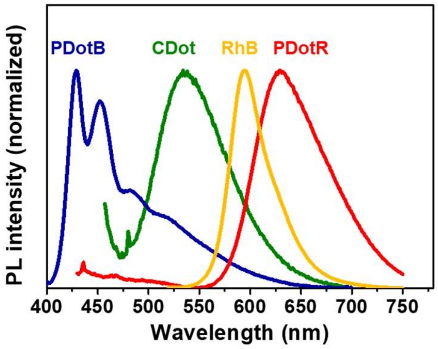

Figure S6. PL spectra of PDotB, CDot, RhB and PDotR solution.
